# Analysis of *znt*A gene in environmental *Escherichia coli* and additional implications on its role in zinc translocation

**DOI:** 10.1007/s13205-017-0613-0

**Published:** 2017-04-08

**Authors:** Anandhan Vidhyaparkavi, Jabez Osborne, Subramanian Babu

**Affiliations:** School of Bio Sciences and Technology, VIT University, Vellore, 632014 India

**Keywords:** *E. coli*, Lead, Translocation, Zinc, *znt*A gene

## Abstract

*Escherichia coli* strains from sewage sample were screened for the presence and expression of heavy metal-translocating *znt*A gene by PCR and RT-PCR analysis with type culture of K-12 as standard strain. The strain which showed high level of gene expression (SBVP1) was chosen to further study the growth and heavy metal translocation. This superior strain was grown in the presence of ZnSO_4_, Pb (CH_3_COO)_2_ and mixture of ZnSO_4_, Pb(CH_3_COO)_2_ metal salts and the growth was observed at different time points. The cell pellet fraction was found to have more of zinc than lead as determined by atomic absorption spectroscopy indicating the translocation of these metals from media to the cells. However, the intracellular translocation of zinc is affected by the presence of lead in the media. Expression of the *znt*A gene in bacteria grown in the presence of ZnSO_4_ was also studied and the molecular analysis results correlate with spectroscopic observations.

## Introduction

Heavy metal pollution causes many genetic problems and has been a great threat around the world. The need for the development of efficient and economic ways for the degradation of these heavy metals is of top priority in the attempts to clean our environment. Use of microorganisms which have metal-resistant capacity in metal polluted areas reduces the metal bioavailability and helps in the bioremediation of the soils (Ji and Silver 1995). These microorganisms possess different types of metal-resistant mechanisms like intra- and extra-cellular metal sequestration, metal efflux pumps, metal reduction and production of metal cheaters (Nies 1992, 1999; Rosen 1996). In the post-genomic era, molecular biological approaches based on genome sequences help to study the different genes and proteins that play a role in the degradation of these heavy metals.

*E. coli,* the most common bacterium found in contaminated soil is known to have many metal degradative properties. The first Zn(II)-translocating P-type ATPase has been identified as the product of *znt*A, a potential gene identified after sequencing of the *E. coli* genome (Rensing et al. 1997). This soft metal-translocating P1-type ATPase confers resistance to Pb(II), Cd(II), and Zn(II).

Although the functional characterization of *znt*A gene in *E. coli* by gene disruption (Rensing et al. 1997; Wang et al. 2012) has been reported, functional analysis in the native *E. coli* isolates of the sewage samples with the aim to screen and identify an efficient bioremediating strain has not been reported so far. In this study, we report the screening and functional characterization of *E. coli* isolates from sewage sample. Several genes and proteins including *znt*A have been reported to play a role in zinc influx and efflux machinery of the bacteria. However, the discrete behavior of the individual genes/proteins under specific conditions, especially when zinc is present along with other heavy metals in the growth media is not studied. We describe here the results of our attempt to study the translocation of zinc when it is present alone as well as in combination with lead in the growth environment of *E. coli.*

## Materials and methods

### *E. coli* strains

Sewage water sample from the sewage treatment plant at VIT University, Vellore, India, was used for the isolation of *E. coli*. Samples were serially diluted and plated on MacConkey and EMB agar. Isolates were subcultured and maintained in EMB agar. *E. coli* type culture strain K-12 (MTCC 1302) was obtained from Microbial Type Culture Collection, Institute of Microbial Technology, Chandigarh, India. Standard biochemical tests viz, Gram’s staining, methyl red, Voges–Proskauer, indole, citrate utilization, triple sugar iron were performed as per the standard procedures.

### Isolation of gDNA from *E. coli* strains

Genomic DNA from *E. coli* isolates as well as the type culture was isolated by CTAB/NaCl procedure. Briefly, the bacterial cells were pelleted down by centrifuging at 8000×*g* for 5 min. The pellet was resuspended in 567 μL of TE buffer, pH 7.5 by repeated pipetting. About 30 μL of 10% SDS and 3 μL of proteinase K stock (10 mg/mL) were added. The suspension was mixed by inverting and incubated at 37 °C for an hour. About 100 μL of 5 M NaCl was added and mixed by inverting. This was followed by addition of 80 μL of CTAB/NaCl solution (10% CTAB, 0.7 M NaCl). Tubes were incubated at 65 °C for 10 min and the contents were extracted with equal volume of chloroform:isoamyl alcohol. After centrifugation at 12,000×*g* for 5 min, the aqueous layer was transferred to fresh tube and 0.6 volume of ice-cold isopropanol was added. DNA was precipitated by centrifuging at 10,000×*g* for 5 min and the pellet was washed with 70% ethanol, air dried and suspended in 20 μL of TE buffer.

### PCR and RT-PCR analysis of *znt*A gene

The sequence of *znt*A gene (*E. coli* K-12 sub strain MG1665 (NCBI GenomeID, NP_000913.2) was obtained from the NCBI microbial genome database and Primer3 online tool was used to design the PCR primers (forward—5′ GCAGGCGTGAATCAGGTGCT 3′ and reverse—5′ GCGCCCGCTGTGATTCGGCG 3′). Primers were synthesized at Bioserve Biotechnologies India Private Limited, Hyderabad, India.

PCR reaction mixture consisted of 100 ng DNA, 0.2 µM each primer, 50 mM KCl, 10 mM Tris Cl (pH 8.3), 1.5 mM MgCl_2_, 200 mM of each deoxynucleotide triphosphates and 1 U *Taq* polymerase. PCR was carried out using following conditions: initial denaturation at 94 °C for 2 min; 30 cycles of denaturation at 94 °C for 1 min, annealing at 52 °C for 1 min, extension at 72 °C for 1 min and a final extension period of 10 min at 72 °C) and analyzed in 1% agarose gel.

Total RNA was isolated from bacterial cell pellet obtained from overnight cultures of the isolates grown in nutrient broth supplemented with 5% v/v sewage water (original source from which isolates were collected) using RaFlex Kit (Genei, Bangalore, India) as per kit instructions. First-strand cDNA synthesis was carried out with 20 ng RNA, AMV reverse transcriptase and reverse primer using cDNA Synthesis Kit (Genei, Bangalore, India) as per the kit instructions. The reaction mixture was incubated at 42 °C for 1 h and terminated at 70 °C for 10 min followed by chilling on ice. PCR was carried out using 5 µl of cDNA (synthesized from 20 ng RNA), 2 U *Taq* polymerase, 200 µM each dNTP and 1.5 mM MgCl_2_. Primers were used at 0.2 µM final concentration. Amplification was done with initial denaturation at 95 °C for 2 min followed by 30 cycles of 95 °C for 1 min, 52 °C for 1 min and 72 °C for 1 min. This was followed by final extension step of 72 °C for 7 min. The PCR products were analyzed in 1% agarose gel stained with ethidium bromide.

### Evaluation of *E. coli* for Zn and Pb tolerance

The *E. coli* isolate which showed high expression of *znt*A gene was used in the study to determine the minimum inhibitory concentration (MIC) of Zn and Pb salts. The isolate was grown in liquid media containing varying concentrations of ZnSO_4_ (0–5 mM) and Pb(CH_3_COO)_2_ (0–5 mM) separately. Heavy metal salt containing media were inoculated with overnight cultures of the isolate and incubated in environmental shaker at 37 °C for 2 days. The growth was measured by recording the OD at 610 nm. The *E. coli* isolate was cultured in liquid media containing MIC of Zn and Pb salt (3.5 and 4 mM, respectively) separately as well as in combination. The cultures were allowed to grow for 5 days and the OD at 610 nm was recorded at a time interval of 24 h.

### Atomic absorption spectroscopy (AAS) analysis of heavy metals

The sewage water used to isolate *E. coli* was analyzed for heavy metals like zinc (Zn), lead (Pb), copper (Cu) and mercury (Hg) at the AAS facility of technology business incubator, VIT University, Vellore, India.

*E. coli* cultures grown in media containing MIC of Zn and Pb salt separately as well as in combination were transferred to 10 mL tubes and centrifuged at 5000 rpm for 10 min. The supernatant was separated. The cell pellet was acid digested using concentrated HCl (Guven and Akinci 2011) by incubating at 90 °C for 2 h. The digested cell extract was filtered through Whatman No. 1 filter paper, diluted and used for AAS analysis. Based on the mean absorbance, concentration of Zn and Pb was determined by comparing with mean absorbance of the standard Zn and Pb.

### RT-PCR analysis of ZnSO_4_-induced *znt*A gene expression

Total RNA was isolated from *E. coli* isolate grown in the MIC of ZnSO_4_ and RT-PCR analysis of *znt*A gene was performed as described earlier. The same isolate grown in media without ZnSO_4_ was used for comparison.

### Results and discussion

The *E. coli* zinc homeostasis is known to be regulated by networking of influx and efflux pumping systems (Hantke 2001; Blencowe and Morby 2003). At high zinc concentration, zntA, a P-type transporter of efflux system is upregulated (Rensing et al. 1997; Outten et al. 1999). In addition, *zit*B, a cation diffusion facilitator (Grass et al. 2001; Lee et al. 2002) and many other transporters are known to play a role in translocation of zinc ions (Grass et al. 2005; Hantke 2005; Wei and Fu 2005). The discrete function of each transporter/facilitator of the influx and efflux system is studied to some extent. Nevertheless, their functions under specific conditions, specifically when zinc is present along with high concentration of other heavy metals are largely unknown. Our study described here represents a step forward in this area of research demonstrating the translocation of zinc when it is present alone as well as in combination with lead in the growth environment of *E. coli.*

**Fig. 1:**
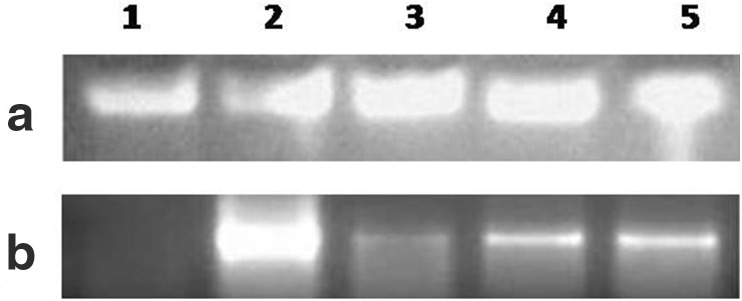
Molecular analysis of *zntA* gene in *E. coli* strains. **a** PCR analysis of *znt*A using genomic DNA isolated from *E. coli* strains; **b** RT-PCR analysis of *znt*A using total RNA obtained from *E. coli* strains. *Lanes 1* K-12, *2* SBVP1, *3* SBVP2, *4* SBVP3, *5* SBVP4

**Fig. 2:**
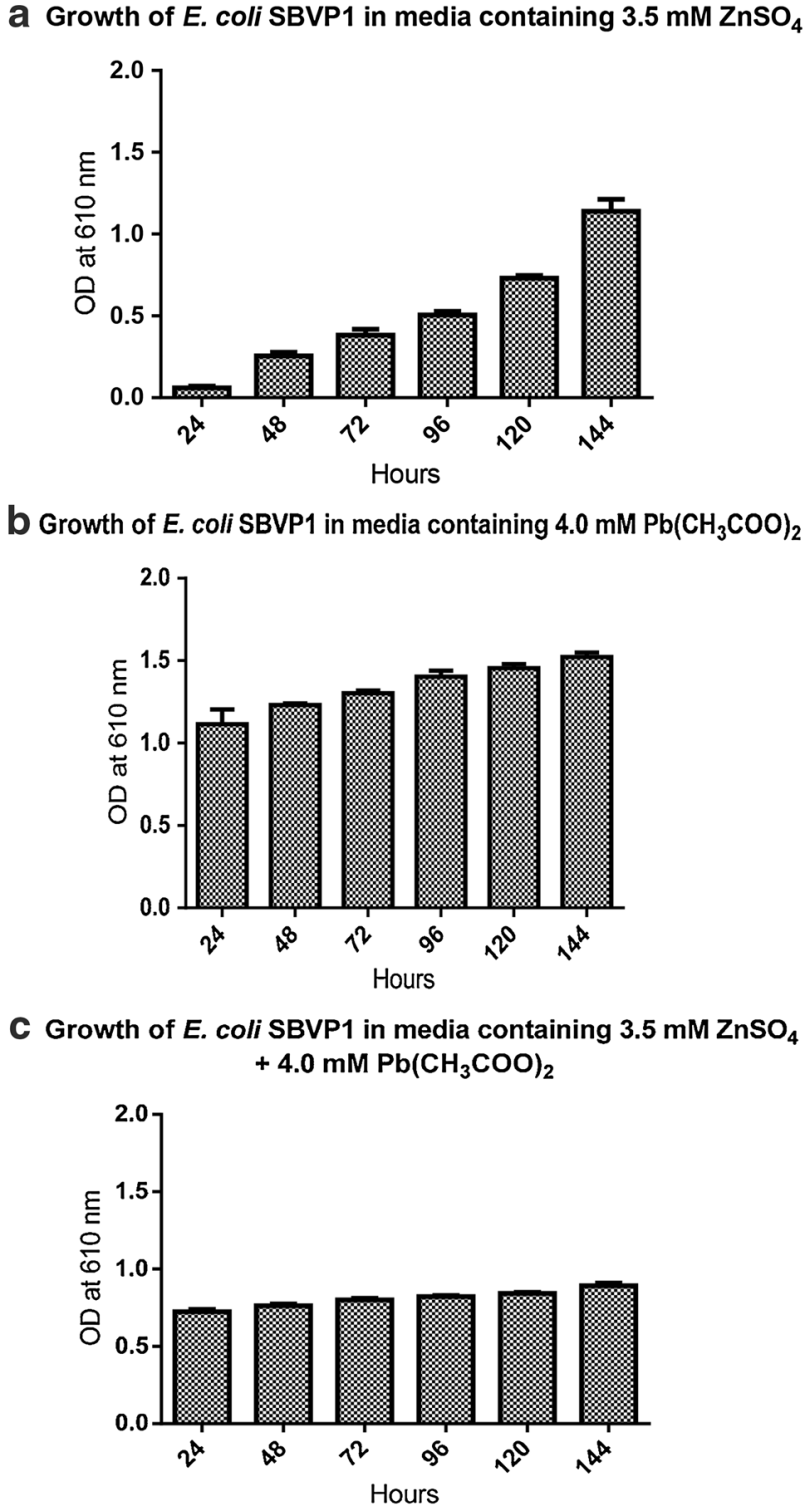
Growth of *E. coli* SBVP1 in media containing Zn and Pb salts separately and in combination

The sewage sample used in the study contained 2.90, 1.04, 0.08 and 0.50 mg/L of Hg, Zn, Pb and Cu, respectively (Table 1). *E. coli* strains were isolated and characterized based on cultural and biochemical methods as described. The strains were designated as SBVP1, SBVP2, SBVP3 and SBVP4. Primers for the amplification of *znt*A gene were designed based on the gene sequence of *E. coli* K-12 substr. MG1655. All the four strains from sewage sample as well as the K-12 type culture showed amplification of the *znt*A gene in PCR done with genomic DNA (Fig. 1a). However, RT-PCR with total RNA obtained from these strains showed expression of the gene in sewage-derived strains only (Fig. 1b). Among these strains, SBVP1 which showed higher expression was chosen for further study and grown in varying concentrations (0–5 mM) of Zn and Pb salts. ZnSO_4_ of 3.5 mM and Pb(CH3COO)_2_ of 4.0 mM were found to be the concentrations after which further increase in concentration resulted in reduced growth of bacteria. The strain SBVP1 was further grown in media containing the optimized concentrations of Zn and Pb salts separately as well as in combination. The results of growth of this strain in this experiment are shown in Fig. 2. The growth was found to be slow but steady in media containing 3.5 mM ZnSO_4_ until observed (144 h). In contrast, the growth of bacteria in Pb salt containing media was high initially but with further flat growth level as compared to steep raise in Zn containing media. When the heavy metal salts were used in combination, a similar flat growth rate was observed. The result of this experiment indicate that the *E. coli* strain although tolerates Zn by possible translocation, the growth is affected by the presence of Pb salt. To confirm these observations, Zn and Pb were estimated in the bacterial cell pellet extract and expressed as mM (Table 2). Cell pellet extract of SBPV1 contained 1.039 mM Zn in ZnSO_4_ containing media compared to 0.308 mM in media containing Zn and Pb salts. The result of this estimation supports the observation made on growth of bacteria. Wang et al. (2012) proposed that the dramatic increase in intracellular free Zn is caused by sudden increase in Zn influx due to increased extracellular concentration coupled with lack of sufficient intracellular ligands to rapidly sequester and buffer all these excess zinc.

**Table 1:** Analysis of heavy metals in sewage sample used in the study

Heavy metal	Standard/sample	Concentration (mg/L)	Mean absorbance
Hg	Standard Hg	15.00	0.0235
	Sewage sample	2.900	0.0045
Zn	Standard Zn	15.00	0.9622
	Sewage sample	1.038	0.0669
Pb	Standard Pb	15.00	0.0703
	Sewage sample	0.080	0.0004
Cu	Standard Cu	15.00	0.3299
	Sewage sample	0.502	0.0110

**Table 2:** Translocation of Zn and Pb by *E. coli* SBVP1

Media supplement	Heavy metal analyzed	Concentration in media (mM)	Concentration in cell pellet after 5 days of *E. coli* growth (mM)
ZnSO_4_	Zn	3.5	1.039
Pb(CH_3_COO)_2_	Pb	4.0	0.038
ZnSO_4_ + Pb(CH_3_COO)_2_	Zn	3.5	0.308
ZnSO_4_ + Pb(CH_3_COO)_2_	Pb	4.0	0.140

**Fig. 3:**
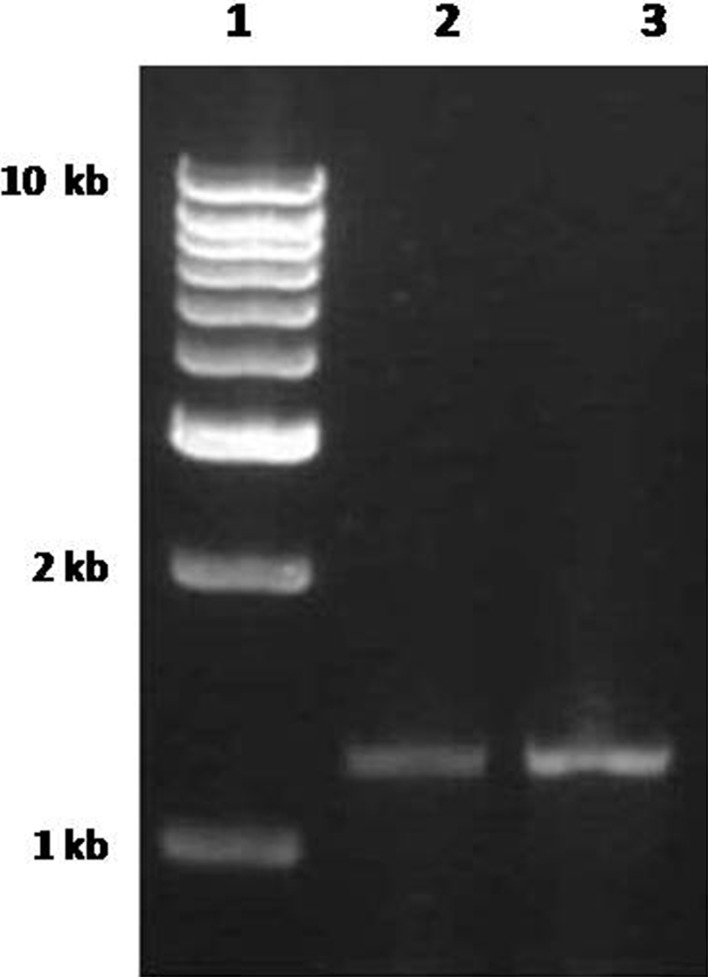
RT-PCR analysis of *znt*A gene expression in *E. coli* (SBVP1) cultured in media containing ZnSO_4_. *Lanes 1* DNA marker, *2 E. coli* SBVP1 grown without any heavy metal in media, *3 E. coli* SBVP1 grown in media with ZnSO_4_

The concentration of Pb in cell pellet extract obtained from Pb + Zn containing media was higher (0.140 mM) when compared to the concentration in cell pellet extract obtained from media with Pb salt alone (0.038 mM). This observation pushes us to an understanding that translocation of Pb require the presence of Zn. In other words, presence of Zn would have triggered *znt*A gene-mediated translocation of Pb. In addition to Zn, *znt*A gene is already known to translocate Pb and Cd (Silver 1996). Moreover, the much higher accumulation of Zn in cells as observed in media containing Zn alone was not the scenario when the media contained Zn and Pb. This observation made us to analyze the expression of *znt*A gene in the cells grown in ZnSO_4_ containing media compared to media without any heavy metal salts. The expression of zntA was higher in the presence of ZnSO_4_ (Fig. 3). The amplified DNA was eluted form the gel and sequenced to confirm the results. The sequence was submitted to GenBank with an Accession no. of KC598125.1.

Gene knock-out studies have shown the elimination of *znt*A resulting in high accumulation of Zn (II) in the cells (Rensing et al. 1997). Furthermore, deletion of *zit*B and *znt*A has proved *znt*A as primary transporter to combat high zinc concentrations (Grass et al. 2001). Wang et al. (2012) employed genetically encoded fluorescent zinc sensor to monitor the intracellular free zinc changes and found increasing expression of *znt*A in a zinc-dependent manner.

We also observed the increased expression of *znt*A in the presence of zinc at high concentration (Fig. 3) which correlated with high level of intracellular zinc in bacterial cells (Table 2). In addition, Wang et al. (2012) also observed prolonged accumulation of zinc in *znt*A mutant *E. coli* and they proposed *zit*B as constitutive first-line defense against toxic zinc influx and the upregulation of *znt*A is to lower the zinc concentration.

In our study, we observed constitutive expression of *znt*A even during normal growth conditions in all the strains of *E. coli* from sewage sample excepting the K-12 type culture. Hence we propose that the presence of zinc in the environmental niche, from which the strains are collected, would have ‘trained’ the *znt*A gene to express constitutively due to continuous exposure to zinc at least in less toxic levels (1.038 mg/L; Table 1). Further analysis of genes like *znt*R, the transcription factor which is known to be activated by zinc and which regulates *znt*A is expected to provide additional evidences towards the aforesaid proposal.

Based on our study we conclude (1) *znt*A is a constitutively expressing gene playing major role in zinc translocation in environmental strains of *E. coli*; (2) higher intracellular accumulation due to zinc shock in growth environment is controlled by the presence of Pb in the same environment; (3) zinc-induced *znt*A gene plays additional role in translocating Pb, intracellularly.
